# Disease registries as foundational infrastructure for Contract Research Organisations in Africa

**DOI:** 10.1017/cts.2026.10243

**Published:** 2026-01-21

**Authors:** Benedict Kusi Ampofo, Fred Stephen Sarfo

**Affiliations:** 1 Directorate of Internal Medicine, Komfo Anokye Teaching Hospital, Ghana; 2 Department of Medicine, Kwame Nkrumah University of Science and Technology, Ghana

**Keywords:** Contract research organisations, disease registries, registry-based trials, clinical trials, Africa

Tilahun and colleagues provide a timely and thoughtful narrative review on the role of Contract Research Organisations (CROs) in strengthening Africa’s clinical trials ecosystem [1]. They clearly articulate the continent’s persistent underrepresentation in global trials, alongside the financial, regulatory, and infrastructural constraints that limit research capacity. Their emphasis on CROs as coordinators of trial logistics, quality assurance, and regulatory compliance is well placed, particularly as sponsor interest in African trial sites continues to grow. We write to extend this discussion by highlighting a foundational element that has received limited emphasis: the role of disease registries and longitudinal data systems as critical infrastructure for CRO effectiveness.

Disease registries constitute the missing infrastructure layer beneath CRO-led trial activity. CROs are designed to optimize trial execution, but their efficiency is structurally dependent on reliable patient identification, longitudinal follow-up, and outcome ascertainment. In many African settings, the absence of harmonized disease registries means that feasibility assessments, recruitment estimates, and endpoint verification are repeatedly rebuilt de novo for each study. This approach inflates costs, prolongs timelines, and increases participant attrition. By contrast, mature disease registries – whether in stroke, cardiovascular disease, cancer, or infectious diseases – create standing cohorts, standardized variables, and continuity of observation that substantially reduce the marginal cost of subsequent trials while improving data completeness and external validity.

Evidence from high-income settings demonstrates the magnitude of this effect. Registry-embedded randomized trials have shown that leveraging existing clinical data platforms can dramatically lower costs, accelerate recruitment, and enhance generalizability without compromising internal validity [2,3]. The TASTE trial, embedded within the Swedish SWEDEHEART registry, randomized over 7000 patients at a substantially lower cost and shorter timeline than a conventional cardiovascular trial, while directly influencing national practice [2,4]. Similar experiences across cardiovascular and other disease registries illustrate that registries are not ancillary tools but core trial infrastructure [2]. In the absence of robust longitudinal disease registries, CRO-led trial activity in many African settings remains episodic, with trial readiness needing to be re-established for each new study rather than sustained over time.

From this perspective, CROs should be conceptualized not only as service providers executing discrete trial functions, but as strategic partners in building and sustaining the data infrastructure required for high-quality clinical research. The prevailing CRO model – largely imported from high-income contexts – positions these organizations as external operators contracted to deliver specific trial functions. In African health systems, sustained trial capacity depends less on one-off service delivery and more on cumulative investment in data systems, governance, and site-level learning. CROs are well placed to support this process by contributing to the development of disease registries, promoting interoperable digital platforms, and aligning trial monitoring and data management with routine clinical information systems. Such an approach allows CRO involvement to generate lasting data assets that support future studies, rather than limiting its impact to the delivery of individual trials.

This shift has direct implications for trial participation and scalability. Disease registries embedded in routine care facilitate earlier and more inclusive patient identification, reduce exclusions related to incomplete records, and support pragmatic trial designs that better reflect real-world practice [5,6]. In addition, registry-linked systems enable post-trial follow-up and real-world evidence generation, aligning with growing regulatory interest in the use of routinely collected healthcare data to support evidence generation alongside traditional trials [7]. CROs that invest in such data infrastructures may therefore strengthen not only operational efficiency, but also the credibility, relevance, and long-term sustainability of clinical research in Africa.

In this context, the model proposed by Tilahun et al. could be further strengthened by explicitly recognizing disease registries and longitudinal data systems as foundational enablers of CRO effectiveness. Efforts to “unlock” CRO potential in Africa should proceed in parallel with investments in registry governance, data harmonization, and interoperability, with CROs incentivized to function as long-term partners in building trial-ready systems. This alignment would support a shift from isolated trial delivery toward cumulative and sustainable research capacity on the continent.
